# Supplementary material for: In vitro induction and identification of polyploid Neolamarckia cadamba plants by colchicine treatment
Source: PeerJ. 2021 Oct 27;9:e12399. doi: 10.7717/peerj.12399 (PMC8556713; doi:10.7717/peerj.12399)
Supplement: Supplemental Information 2 [file peerj-09-12399-s002.docx]

**ANOVA TABLE**

**Table S1** Statistical analysis of shoot regeneration, growth, rooting and acclimatization of tetraploid, mixoploid and octoploid of *N. cadamba*

| **Parameters** | **DF** | **Sum of**  **Square** | **Mean**  **of Square** | **F value** | **Pr > F** | **Eta-squared** |
| --- | --- | --- | --- | --- | --- | --- |
| **Regeneration** |  |  |  |  |  |  |
| Number of shoots ± SE | 2 | 2.397 | 1.198 | 9.173 | .015 | 0.754 |
| Regeneration (%) ± SE | 2 | 432.272 | 216.136 | 7.000 | .027 | 0.700 |
|  |  |  |  |  |  |  |
| **Growth** |  |  |  |  |  |  |
| Stem height (cm) ± SE | 2 | 13.645 | 6.823 | 2741.268 | .000 | 0.999 |
| Number of leaves ± SE | 2 | .075 | .037 | .381 | .699 | 0.113 |
| Number of nodes ± SE | 2 | .018 | .009 | .364 | .709 | 0.108 |
| Stem diameter (mm) ± SE | 2 | .466 | .233 | 10.898 | .010 | 0.784 |
|  |  |  |  |  |  |  |
| **Rooting** |  |  |  |  |  |  |
| Number of roots ± SE | 2 | 38.379 | 19.190 | 12.318 | .008 | 0.804 |
| Root length (cm) ± SE | 2 | 0.288 | .144 | 32.420 | .001 | 0.915 |
|  |  |  |  |  |  |  |
| **Acclimatization percentage** |  |  |  |  |  |  |
| Tetraploid | 2 | 3518.259 | 1759.130 | 11.395 | .009 | 0.792 |
| Mixoploid | 2 | 2221.778 | 1110.889 | 11.993 | .008 | 0.800 |
| Octoploid | 2 | 1295.815 | 647.907 | 10.492 | .011 | 0.778 |
|  |  |  |  |  |  |  |

**Table S2** Statistical analysis of morphological characteristics and SPAD value

| **Parameters** | **DF** | **Sum of**  **Square** | **Mean**  **of Square** | **F value** | **Pr > F** | **Eta-squared** |
| --- | --- | --- | --- | --- | --- | --- |
| **Leaf thickness** |  |  |  |  |  |  |
| Leaf thickness (mm) ± SE | 2 | .038 | .019 | 3.170 | .058 | 0.190 |
| Midrib vertical thickness (mm) ± SE | 2 | .137 | .069 | 3.508 | .044 | 0.206 |
| Midrib horizontal thickness (mm) ± SE | 2 | .408 | .204 | 8.431 | .001 | 0.384 |
|  |  |  |  |  |  |  |
| **Stomata and trichomes** |  |  |  |  |  |  |
| Stomata density (mm^-2^) ± SE | 2 | 107029.051 | 53514.526 | 65.171 | .000 | 0.828 |
| Trichomes density (mm^-2^) ± SE | 2 | 5641.862 | 2820.931 | 31.610 | .000 | 0.701 |
| Stomata length (mm) ± SE | 2 | .002 | .001 | 80.711 | .000 | 0.352 |
| Stomata width (mm) ± SE | 2 | .000 | .000 | 16.118 | .000 | 0.098 |
|  |  |  |  |  |  |  |
| **Stem cross-section** |  |  |  |  |  |  |
| Bark (mm) ± SE | 2 | .000 | .000 | .689 | .538 | 0.187 |
| Wood (mm) ± SE | 2 | .009 | .004 | 1.515 | .293 | 0.336 |
| Pith (mm) ± SE | 2 | .071 | .035 | 18.775 | .003 | 0.862 |
|  |  |  |  |  |  |  |
| **SPAD value** |  |  |  |  |  |  |
| SPAD ± SE | 2 | 41.253 | 20.626 | 4.263 | .024 | 0.240 |
|  |  |  |  |  |  |  |
